# Supplementary material for: Comparative multiomics analysis of cell physiological state after culture in a basket bioreactor
Source: Sci Rep. 2022 Nov 23;12:20161. doi: 10.1038/s41598-022-24687-4 (PMC9686226; doi:10.1038/s41598-022-24687-4)
Supplement: Supplementary file 1 — Supplementary Information 1. [file 41598_2022_24687_MOESM1_ESM.zip › raw data/Transcriptomic raw data/6.Enrichment/2.KEGG/TE1vsTC1/ALL/src/csab00100.html]

csab00100


- csab:103226567

- Up regulated genes

103226567(1.68268521155497)

- csab:103226567

- Up regulated genes

103226567(1.68268521155497)

- csab:103215320

- Up regulated genes

103215320(1.22102475929591)

- csab:103215320

- Up regulated genes

103215320(1.22102475929591)

- csab:103237411

- Up regulated genes

103237411(1.61512850099932)

- csab:103226567

- Up regulated genes

103226567(1.68268521155497)

Close
